# The Common Form of Dysmenorrhœa of Young Women

**Published:** 1907-11-02

**Authors:** 


### Gynæcology.

### DYSMENORRHŒA IN YOUNG PATIENTS.

In young women, virgins, or married women who have had no children, the cause of painful menstruation is often exceedingly obscure, and although the term "spasmodic" fits the cases best, it does not in the least suggest what is their causation. In this sense it is a useful term, and much better than the older term "obstructive dysmenorrhœa." The latter term must be reserved for a very small group of cases in which the cervix is actually stenosed, usually as a result of an operation such as amputation of the cervix. The common type is familiar to all who have any experience of family practice. The girl who is seized with pain in the lower part of the abdomen a few hours before, or just as, the menstrual flow begins, accompanied sometimes by nausea or vomiting, and then after a continuance of pain for a few hours has a violent headache and recovers in a day or two is a very familiar patient. As a general rule this kind of dysmenorrhœa is primary—that is, it begins when menstruation is first established—but it is apt to get worse as the patient gets older. In some happier cases the pain gets less as time goes on, and occasionally disappears with adult life. The pain in these cases is truly spasmodic—that is, it is of an intermittent character—coming and going, and essentially commencing with the flow or just before it. In this sense it irresistibly recalls the characters of "colicky" pains of intestinal origin. It was this colicky character which led the older gynæcologists to believe that it was the result of uterine contractions attempting to expel the uterine contents against an unusual resistance. Herman and others have proved, however, that no obstruction really exists in most of these cases. Where clots occur in the menstrual products, it is more than probable in most instances that these form in the vagina, and not in the uterus, and, therefore, it cannot be that the expulsion of clots is the cause of the pain. Also it is quite unusual for any clots to be constantly present in these cases. The view which best explains them is that elaborated by Theilhaber, on the supposition that the pain in these cases is really due to spasmodic or irregular uterine contractions, due to imperfect development of the uterine muscle. There is no difficulty in this theory, first because of the character of the pain, and secondly because the uterus has to contract at all times to expel its contents whether menstruating or not. The effect of the absence of contractions is well shown in the subjects of senile endometritis, when the inflammatory products, usually pus, collect in the uterus because there are no contractions to expel them owing to muscular degeneration. These cases, to which the term pyometra has been given, occur when there is no obstruction to the outflow, the cervix being quite patent. A further testimony to the probability of this theory is offered by the process of natural cure—namely, by pregnancy. Pregnancy cures these cases for two reasons, first because pregnancy leads to the greatest amount of development of the uterine muscle, and secondly because pregnancy leads to the natural stretching of the lower uterine segment to its utmost capacity. Unfortunately marriage cannot be confidently relied upon to bring about this cure, because the patients often do not conceive readily. The reason for this is not always obvious, but it is commonly believed to be in some way connected with the shape of the uterus and its state of development. Although no fixed type of uterus can be said to be present in all cases of this kind, it is quite common to find an undue amount of anterior flexion, combined with a long conical cervix. In other words, a malformed, not a displaced, uterus in which it is not improbable that the muscle coats are poorly developed along with the other congenital malformation. However, it is not uncommon to find that these patients have small retroverted uteri, the displacement being congenital and having nothing to do with the menstrual anomaly; the small size really represents a maldevelopment and is the actual cause of the pain. It would be idle to suppose that congestion of the uterus plays no part in this condition, because it is clear that it must do. The important point, however, is that the præmenstrual congestion of itself is not sufficient to produce pain until contractions appear. Even this statement is not absolute, because sometimes there is a constant ache (due to congestion) before the spasmodic pains begin. If the causation of the cases is often obscure the treatment of them is more than ordinarily difficult. It is usual to commence by treating such cases in a general way by attention to the bowels, digestion, circulation, and general hygiene. This is often all the treatment that is necessary; the anæmic constipated girl who has dyspepsia in addition, almost always has menstrual pain, which is cured if these disabilities are relieved. It is also true that no treatment directed to the local condition is likely to do good until these other troubles are relieved; after that we are justified in treating the pain itself. Let it be clearly realised that there are some drugs which must not on any account be given, namely morphia and alcohol. Gin is the time-honoured treatment, and nothing is easier than to give a small dose of morphia, but really both are most pernicious for obvious reasons. The antispasmodic drugs, antipyrin, phenacetin and cannabis indica all do good to some patients, the most generally useful one being antipyrin, whilst acetyl-salicylic acid will sometimes give relief where others fail. There is a limit, however, beyond which these drugs will not go, and then an operation is indicated. Simple dilatation of the cervix with graduated sounds is but begging the question, especially if imperfectly done without an anæsthetic. Dilatation must be carried out to the full degree, about 14 to 16 Hegar, under an anæsthetic with most rigid antiseptic precautions, and if the cervix is long and conical it may be slit backwards at the same time, sutures being put in to prevent the cut edges adhering. Although opinions differ on the point, thorough curettage after this dilatation has in our experience given better and more lasting results than simple dilatation. The whole operation, however, must be rigidly aseptic.

